# Characterization of Metal Powders Used for Additive Manufacturing

**DOI:** 10.6028/jres.119.018

**Published:** 2014-09-16

**Authors:** JA Slotwinski, EJ Garboczi, PE Stutzman, CF Ferraris, SS Watson, MA Peltz

**Affiliations:** National Institute of Standards and Technology, Gaithersburg, MD 20899

**Keywords:** additive manufacturing (AM), Direct Metal Laser Sintering (DMLS), energy-dispersive x-ray, helium pycnometry, laser diffraction, metal powder, powder bed fusion, X-ray computed tomography, X-ray diffraction, scanning electron microscopy, X-ray photoelectron spectroscopy

## Abstract

Additive manufacturing (AM) techniques^1^ can produce complex, high-value metal parts, with potential applications as critical parts, such as those found in aerospace components. The production of AM parts with consistent and predictable properties requires input materials (e.g., metal powders) with known and repeatable characteristics, which in turn requires standardized measurement methods for powder properties. First, based on our previous work, we assess the applicability of current standardized methods for powder characterization for metal AM powders. Then we present the results of systematic studies carried out on two different powder materials used for additive manufacturing: stainless steel and cobalt-chrome. The characterization of these powders is important in NIST efforts to develop appropriate measurements and standards for additive materials and to document the property of powders used in a NIST-led additive manufacturing material round robin. An extensive array of characterization techniques was applied to these two powders, in both virgin and recycled states. The physical techniques included laser diffraction particle size analysis, X-ray computed tomography for size and shape analysis, and optical and scanning electron microscopy. Techniques sensitive to structure and chemistry, including X-ray diffraction, energy dispersive analytical X-ray analysis using the X-rays generated during scanning electron microscopy, and X-Ray photoelectron spectroscopy were also employed. The results of these analyses show how virgin powder changes after being exposed to and recycled from one or more Direct Metal Laser Sintering (DMLS) additive manufacturing build cycles. In addition, these findings can give insight into the actual additive manufacturing process.

## 1. Introduction

Unlike traditional manufacturing processes such as turning and milling that produce parts by removing unwanted material from a larger piece, additive manufacturing (AM) processes build parts one thin layer at a time. This can be done in a variety of ways, such as melting or sintering of powder via laser or electron beams, extrusion and deposition of polymer via a heated orifice, or selective curing of liquid photopolymers. These processes can all produce complex, high-value parts with internal features that cannot be fabricated with traditional material removal processes, can accomplish this without tooling, and with the ability to go almost directly from a digital design to part. The full vision of additive manufacturing includes using these processes to produce complex, customized metal parts for use in high-stress, mission-critical aerospace applications, such as jet engine components and turbine blades, where innovative, weight-saving part designs that include complex interior structures could revolutionize the manufacturing industry.

Additive manufacturing successes have received significant media attention in the popular press in the last couple of years, and while additive manufacturing is already producing customized metal parts in niche applications such as dental implants [1], and demonstrating truly impressive capabilities that generate lots of societal excitement for the future of AM [2,3], the full benefits of additive manufacturing are not yet realized in a widespread way across the manufacturing industry. This is due in part to a lack of AM-specific standard methods for characterizing both the raw powders used as input materials and for characterizing the mechanical properties of the finished parts [4,5]. Determining the properties of powder used in a metal-based AM system is a necessary condition for industry to be able to confidently select powder and produce consistent parts with known and predictable properties. This paper examines the characterization of metal powders; a companion paper characterizes the mechanical properties of metal AM parts [6].

Consistent powder characteristics are important for ensuring repeatable manufacture of metal parts. For example, metal powders used in AM are assumed to be nominally spherical, and have a particle size distribution that is designed to facilitate good packing behavior, such that the final manufactured part has good mechanical properties and is fully dense. Other characteristics include morphology, density, chemical composition, flow, and thermal properties. In this paper we summarize the applicability of existing standardized powder measurement methods for AM powders, and apply several sophisticated metrology methods to measure the size distribution, morphology, density, and chemical composition of AM metal powders. These methods are used to (1) determine the variability of powders from nominally identical batches, since when different batches of powder from the same lot are used it is assumed that the powders are identical; (2) determine the impact of recycling on powder characteristics; and (3) characterize powder used in recent NIST-led inter-laboratory studies for developing the procedures needed to determine design-allowable mechanical properties of AM materials. This work is also necessary for future studies that will correlate AM powder properties with the mechanical properties of parts made via AM.

## 2. Metrology Methods Employed In This Paper

In this paper commercially available stainless steel (17-4SS) and cobalt chrome (CoCr) powders were characterized using laser particle size distribution (PSD) measurements, X-ray computed tomography (XCT) accompanied by spherical harmonic (SH) analysis, X-ray diffraction (XRD) and scanning electron microscopy (SEM) accompanied by energy dispersive elemental analysis (EDS), and X-ray photospectroscopy (XPS). The laser PSD measured particle size, as defined by the instrument, the XCT measured various particle size and shape parameters and gave three-dimensional (3-D) images, XRD measured particle crystalline phases, SEM gave quasi-3-D images at magnifications and resolutions significantly better than did XCT, and XPS gave a measurement of chemical state of and concentration of elements on the powder surface. Although there is some overlap between techniques in terms of measurands, this overlap serves to better compare the instruments and together they give a fairly complete picture of the size, shape, and composition of the metal powders, even after recycling and reuse. Table 1 summarizes the methods and their associated measurands.

### 2.1 Helium Pycnometry for Particle Density Measurements

The first step was to measure the true density of the metal powders. This number is essential to determine built part porosity since the ratio of that density to the metal powder density directly gives the porosity. This is the approach used in NIST determinations of porosity in a series of CoCr samples described elsewhere [7,8]. Since the metal powder particles are tens of micrometers in size, it would be difficult and slow to measure the volume and mass of individual particles. Helium pycnometry [9,10] can be used to measure the true density of the solid backbone of a porous material, as long as all the pores are accessible by helium gas flowing through the sample. A loose powder compact, like that measured here, should have accessible pores where the assumption is that the particles are fully dense so that the only pores are between the particles. If the particles themselves have internal porosity that is inaccessible from the particle surface, this porosity will be factored into the density determination of the particles. In all the imaging techiques described in this paper, no internal particle porosity was observed.

This technique works by measuring the actual volume of the particles themselves in a sample of powder. A simple mass measurement of this quantity of powder will then give the density by dividing the mass by the volume. A sample of powder that has been carefully weighed is placed into a pycnometry container of known volume. Helium, under controlled temperature and atmospheric pressure, flows into the container, displacing the air. The volume of helium filling the container is known using the ideal gas law, and subtracting it from the container volume gives the volume of the particles.

### 2.2 Laser Diffraction for Particle Size Measurement

A method used to measure the particle size distribution of metal powders is Laser Diffraction (LD). The LD method involves the detection and analysis of the angular distribution of scattered light produced by a laser beam passing through a dilute dispersion of particles [11]. The total scattering or diffracted light pattern is mathematically inverted to give a particle size distribution of spheres that would give the equivalent scattering pattern. The surface area is calculated from the diameter distribution of the spherical particles. In general, the LD method requires that the particles be dispersed, either in liquid (suspension) or in air (aerosol). The former is commonly referred to as the “wet” method (LD-W), while the latter is termed the “dry” method (LD-D).

For the stainless steel and cobalt chrome powders examined in this study (see Sec. 3.1), the LD-W method was used to ensure complete dispersion and minimize the danger of powder ignition. The LD method is widely used for many different kinds of particles across many different industries [12]. A standard test method exists for metal powders, ASTM B822-10 [13]. The medium used in the tests performed was isopropanol alcohol (IPA). The procedure adopted requires that after the background is measured, the powder is measured 25 times after sonication to ensure complete dispersion. Then, three sets of six measurements are performed. Averages are calculated for each set and compared. If these three averages are not consistent, it is assumed that the dispersion of the powder in the IPA was not complete, and more tests are performed. The test data is then analyzed using the Mie theory, which requires the real and imaginary refractive indices of the particles [14]. However, since the particles in this study are generally larger than 7 μm, simple Fraunhofer theory can be used to calculate the distribution, which does not require the particle material refractive indices to be known [13,15]. Both Mie and Fraunhofer theories were used for both sets of particles, and no appreciable difference was seen. This procedure is essentially exact for spherical particles, but is only an approximation for non-spherical particles, which are reported in terms of equivalent spheres that have the same average scattering pattern.

The main advantage of the LD method is that it is fast, and if proper dispersion is achieved, repeatable. The main disadvantage is that the mathematics behind the LD method assumes spherical particles. For the metal powders, this is a fairly reasonable assumption, as can be seen in the SEM and X-ray CT images to be presented later. The particles appear to be nearly spherical, but with notable exceptions.

### 2.3 X-Ray Computed Tomography

In X-ray computed tomography (CT), X-ray radiographs from hundreds of different angles are taken of a sample. Contrast comes from differences between the X-ray attenuation characteristics for each kind of material. Mathematical algorithms use this information to reconstruct the interior of the sample. Output is in the form of hundreds or thousands of cross-sectional images, which can be stacked together to form a 3-D image of the sample. A special procedure for acquiring and analyzing star-shaped particles via a combination of X-ray CT and spherical harmonic series was used [15,16]. “Star-shaped” is a weaker condition than convexity and essentially means that there is a point in the interior of the particles from which a line segment can be drawn to the exterior by only crossing the particle surface once [15,16]. The metallic powders were embedded in 3 mm diameter epoxy cylinders, and images were taken at a pixel size of about 2 μm. The images were 2000 × 2000 pixels square, or about 4 mm × 4 mm. Once the slices were assembled into a 3-D structure, a gray scale threshold was applied to make the particles white and the background black. Special software extracted the particles and analyzed their shape using spherical harmonic series, which gave a mathematical representation for each star-shaped particle. The particles that were judged by the software to not be star-shaped were usually two or more particles stuck together, either in reality or because of the thresholding.

A goal of this current work is to show how different samples taken from nominally identical powder may differ from each other in their shape and size distribution. The size distribution was also measured by laser diffraction, but the CT gives an independent estimate and uses different size parameters to measure the PSD. However, it uses fewer particles, so the particle size distribution curves are generally not as smooth. The SEM gives detailed images of the particles, and can give some degree of perspective also, so that the images can be quasi-3D. But the X-ray CT gives direct, 3-D shape information about the particles. This particle shape is the focus of this section.

To make samples for both the 17-4SS and CoCr powders, a dilute suspension of the particles was created in a marine epoxy [16]. The volume fraction of powder in the suspension varied, but was usually only a few percent by volume. After vigorous stirring, to make sure that the particles were suspended, a laboratory vacuum line was used to draw up the suspension into a plastic straw of about 2 mm inner diameter and length about 12 cm. The straw was capped at both ends with putty, put into a 4 cm diameter cylinder of polymer insulating rigid foam, and then rotated continuously while curing to minimize powder settling effects in the epoxy. After cure, the ends were cut off and the sample was divided into a larger and a smaller piece. Usually the larger piece by itself gave enough particles for analysis. The larger sample piece was put upright into an aluminum sample holder and placed into a commercial X-ray CT machine. All samples were scanned at about 2 μm per voxel. A typical sample gave 6000 slices, where each slice was 2000 × 2000 pixels. The number of angles used was 1440, and exposure times within the CT were less than a second for each of the 1440 images.

Using image analysis software, a reasonable threshold that gave binary images of white particles on a black background was used. The slices were then assembled into a 3-D microstructure and stored in a file as 2-byte integers. Special software was used to identify and isolate each cluster of white voxels. If the number of voxels was too small, generally less than 512, then the particle was not further analyzed. This number of voxels has been determined to be roughly the minimum needed to get accurate shape information from the particle [17]. The cube root of 512 is 8, which, if multiplied by the average voxel size of 2 μm per voxel, means that particles of size roughly 16 μm or less were not analyzed for shape. Because of the manufacturing method used for the particles, a dependence of shape on size was not expected in this size range. Nonetheless, the laser PSD results indicated that there were very few of this size or smaller particles.

If the voxel volume was greater than 512, a series of checks was done to approximately eliminate artificially touching particles. A spherical harmonic series was fit to the perimeter of the particle and the volume computed. If the volume as calculated from the spherical harmonic fit was within 3 % of the voxel volume, then the particle was saved. Having a volume difference greater than 3 % usually means that the spherical harmonic series missed part of the particle, which is almost always due to being a multi-particle conglomerate. This is another automatic error correction. Since some of the samples definitely had multiple-particle conglomerates, another program was run to save only these particles and make 3-D Virtual Reality Modeling Language (VRML) images of the voxel clusters representing these particles.

The end result is a list of particles, each individually numbered, with spherical harmonic coefficients saved in a file with the same number, which can be used to create a VRML 3-D image or compute any integral over the volume or surface of the particle. The particles that were saved were then analyzed further, using their spherical harmonic series, and various geometric quantities were computed, including volume, surface area, integrated mean curvature, length, width, and thickness, the volume equivalent spherical diameter, and the moment of inertia tensor. The volume equivalent spherical diameter (VESD) is defined as the diameter of the sphere that has equal volume to a given particle. If the particle is an exact sphere, then the VESD is equal to the actual diameter of the particle. The integrated Gaussian curvature was also computed. This value, which is derived by integrating the Gaussian curvature, defined at each surface point, over the particle surface using the spherical harmonic expansion, is normalized by 4π and must be equal to unity for a star-shaped particle with a closed surface. This fact was also used as an error check for the particles analyzed [17].

### 2.4 X-Ray Diffraction (XRD)

X-ray diffraction analysis is used for qualitative and semi-quantitative determination of the crystal forms of materials. X-ray diffraction was used to determine which crystalline phases are present, by examining the respective diffraction patterns, displayed as diffraction peaks, which are unique for different compounds. Phase identification is usually made by employing the International Centre for Diffraction Data diffraction database^2^, which catalogues the X-ray diffraction patterns of thousands of crystals. ASTM E975 is a standard practice for the determination of austenite in steel using X-ray diffraction [18]. If more than one crystalline phase is present, Rietveld analysis [19–21] of the diffraction data can provide quantitative estimates of the phase abundances in terms of relative volume fraction. Rietveld analysis is difficult to perform for steel alloys, so the relative phase abundance results reported for the 17-4SS powder are at most semi-quantitative. The CoCr powders seemed to consist of a single-crystalline phase.

X-ray diffraction was used to determine which crystalline phases were present in the powder materials and their approximate proportions. A cavity mount approximately 12 mm × 20 mm and 800 μm deep was carefully filled with grains of powder and the surface leveled with the specimen holder surface. Usually, powder diffraction experiments require a specimen in a fine powder form, with particle size less than 5 μm to improve specimen homogeneity. The coarse, uniform to bimodal particle size, and spherical nature of the grains result in a less than ideal specimen surface with porosity and a relatively rough texture. However, processing the samples to form a more ideal specimen surface (flat, solid) is likely to affect the materials by the pressure and heat of machining. The powders were studied as-is without any reduction in particle size, resulting in surface-sensitive measurements. For a typical Fe-Cr-Ni stainless steel, the bulk of the X-ray signal is emanating from depths approximately up to 4 μm. The individual crystals, as observed by electron microscopy, appear to be micrometer-sized grains so the data represent a surface layer of a few crystal grains thickness. However, the shallow interaction volume may provide an advantage in that changes in the surface material characteristics may be more easily detected. For both sample sets, three replicate scans using quantitative XRD were performed to provide identification of the crystalline forms and estimates on the mass proportions.

### 2.5 Scanning Electron Microscopy with Energy Dispersive Elemental Analysis

In a scanning electron microscope, a rastering electron beam, instead of light, is used to form an image. The images taken here were in secondary electron mode, where low-energy electrons scattered by the particle surfaces form high-resolution images of surface topography. X-rays characteristic of the material chemistry are generated as a result of the electron beam-specimen interaction, and provide a qualitative analysis of the bulk chemistry and, with appropriate instrument calibration, quantitative bulk chemistry. The interaction volume for X-ray microanalysis is dependent upon the accelerating voltage utilized in the imaging and is approximately 1 μm in steel at 15 kV accelerating voltage, making these analyses sensitive to a depth of approximately one crystal layer. Accurate quantitative analysis requires a flat, polished surface and appropriate reference standards for calibration.

### 2.6 X-Ray Photospectroscopy

X-ray photoelectron spectroscopy (XPS) is a surface-sensitive analytical tool that provides information about the chemical state and concentration of elements from the outmost layers (< 10 nm) of a solid material [22,23].

All elements, except hydrogen and helium, can be detected with a detection limit of < 0.1 atomic percent. Photoelectrons are emitted from the solid when it is exposed to a flux of X-ray photons of known energy, *hv*, where *h* is Plank’s constant and *v* is the photon frequency. The photoelectrons come from discrete electron energy levels associated with atoms in the analysis volume. The kinetic energy (*KE*) of the emitted photoelectrons and hence all photoelectron spectroscopy is expressed by the Einstein photoelectric law, *KE* = *hv − BE*, where *BE* is the binding energy of the particular electron to the desired atom. Since *hv* is known, a measurement of *KE* determines *BE*. Since ionization may occur in any shell for a particular atom, the spectrum for that element is unique and composed of a series of peaks corresponding to electron emission from the different shells. This allows for unequivocal elemental identification, since the energy separation and relative intensities of the peaks for a given element are well known. Additionally, ionization for p, d, or f shell levels leads to doublet structures in the spectrum as a result of spin-orbit interactions. Therefore, elements with higher atomic numbers have peaks reflecting the spin-orbit energy separations. Many of these transitions are characteristic of the element in a particular oxidation state, which is of particular interest for powder surfaces that have been exposed to oxygen in the environment and nitrogen and other gases at high temperature during the additive manufacturing process. There is also a dependence of the core level *BE* on the oxidation state and/or local electronic environment about the desired atom. These core electrons are strongly affected by the valence electron distribution and the variations in *BE* are referred to as chemical shifts.

XPS requires ultra-high vacuum instrumentation. The sample area examined is small and can range from 70 μm^2^ to 1 cm^2^. Certain materials are sensitive to surface photoreduction and ion beam damage effects [22]. In this paper, XPS measurements were performed with a commercial system (base pressure: 1.3 × 10^−6^ Pa; Al Kα x-ray: 40 W (14 kV, 10 mA); no coaxial charge neutralization needed for the metal powders; analysis area: 2 mm × 1 mm). Powder specimens were mounted on the multiple sample bar using SEM carbon tape; residual powder was removed before insertion into the instrument.

## 3. Results

### 3.1 Powder Samples Examined

Two types of metal powders, made via gas atomization and used in a commercial laser powder bed direct metal laser sintering (DMLS) additive manufacturing system, were examined in this study:

- Samples from four different containers of nominally identical, virgin 17-4^3^ stainless steel powders (17-4 SS) [25], all from the same production heat lot. These samples were examined to determine potential variability in the properties of powders taken from the same production heat lot.
- Samples from 15 different containers of nominally identical, virgin Cobalt Chromium powders (CoCr) [26], all from the same production heat lot, for use in a NIST-managed AM material round robin study. These samples were examined to determine potential variability in the properties of powders taken from the same production lot.
- Samples of 17-4 stainless steel powder, in virgin form, and recovered after each of eight different builds, both sieved and unsieved. These samples were taken to determine the changes in the powder properties as a function of the number of times the powder is recycled.
- One sample of 17-4 stainless steel sieve residue; reclaimed powder from an AM build that had powder particles that were too large to sift through the 80 μm sieve employed for recycling powder for future builds.

Note that throughout this paper the terms “containers” and “samples” are used interchangeably, with sample #1 coming from container #1, etc. All powder samples were taken from the containers using industry accepted sampling techniques [24].

### 3.2 Density

Helium pycnometry, using a commercial instrument as described in Sec. 2.1, was used to measure the density of the metal powders, which is assumed to be the density of a fully dense built part that has no discernible porosity. Details of the technique, not previously given, are first described. An empty container was used to tare a mass balance. The metal powder was added to fill the cell, lightly tamped, and the mass of the powder determined. In the helium pycnometer, the amount of helium that fills the empty volume around the powder is determined by using the measured temperature and pressure of the helium in the cell and the ideal gas law, which is very accurate for helium at room temperature and pressure. Since the empty cell volume is precisely known, by using the pycnometer on the empty cell, the difference between the two volumes is the actual volume of the powder. A simple quotient gives the powder density, averaged over all the particles present. As was mentioned earlier, if some of the particles are porous, but the pores are accessible from the surface, then the true metal density is still determined. If there are hollow particles such that some pores in the particles are not accessible from the surface by the helium atoms, then these pores will be considered part of the powder and thus the determined powder density will be somewhat smaller than the actual metal density. No evidence for hollow particles was seen with any of the imaging techniques used in this paper.

For the two kinds of metal powders, one run took approximately 30 min. Judging from past experience with other kinds of powders that did have internal porosity, as seen by SEM and X-ray CT, if the metal particles had significant internal yet surface accessible porosity, the run time would have been longer for the helium to penetrate the particles.

Table 2 gives the results for different measurements of the 17-4 SS and CoCr powders. The maximum variation among the results is 0.004 g/cm^3^. The estimated uncertainty in the density measurement (one standard deviation) combines the container volume uncertainty (0.02 % of a 1 cm^3^ volume), which dominated the much smaller mass measurement uncertainty (0.0001 g). Together, these two sources of uncertainty give a 2σ result of about 0.005 g/cm^3^, in agreement with the variation seen in the several experimental density measurements. These values also agree well with literature and manufacturer values for these materials, which, to two significant figures, are 7.8 g/cm^3^ and 8.3 g/cm^3^, for the 17-4 SS and CoCr, respectively [25,26].

### 3.3 Variability of Nominally Identical Containers of Powder

#### 3.3.1 Laser Diffraction

**Fig. 1:**
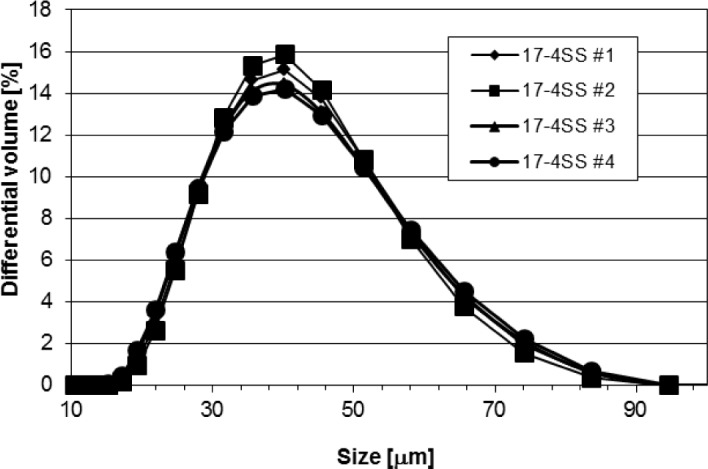
The differential PSD curves for the four 17-4SS samples.

**Fig. 2:**
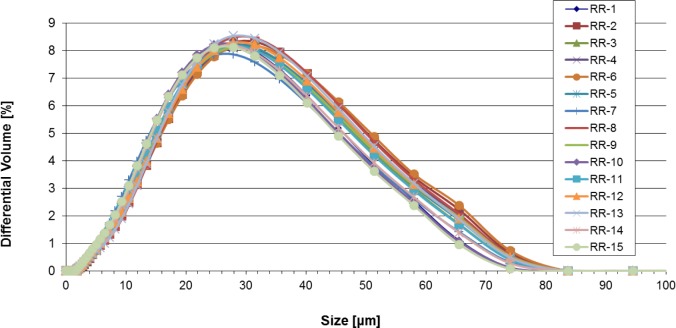
Differential PSD curves for the fifteen CoCr samples.

**Fig. 3:**
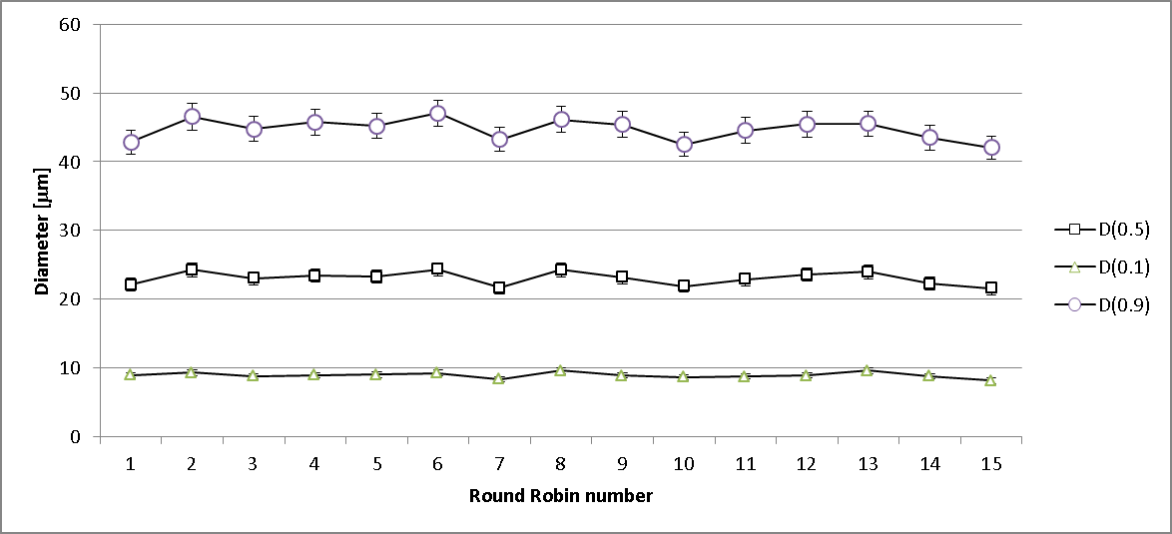
D(0.1), D(0.5), and D(0.9) results for each of the 15 CoCr containers examined.

Figures 1 and 2 show the particle size distribution (PSD) curves for the 17-4 SS and CoCr samples; Tables 3 and 4 present the corresponding data. The various PSD curves overlap each other, implying that the samples for each material were identical within experimental uncertainty (estimated to be well less than 1 % for the LD PSD curves). To examine this point more quantitatively, D(α), where D is the diameter, as measured by LD, for which α is the mass or volume percent of particles with measured diameter less than D, was calculated. For the four 17-4 SS samples, D(0.1) = 24.4 μm ± 0.6 μm, D(0.5) = 36.5 μm ± 0.2 μm, and D(0.9) = 54.5 μm ± 0.8 μm, where the uncertainty is one standard deviation calculated from the four distinctive samples. For the 15 CoCR vials, D(0.1) = 8.9 μm ± 0.4 μm, D(0.5) = 23.0 μm ± 1.0 μm, and D(0.9) = 44.7 μm ± 1.5 μm, where the uncertainty is one standard deviation calculated from the fifteen results. The CoCr powder is smaller, on average, than the 17-4 SS powder, and the size range of the CoCr powder extends to significantly smaller diameter values than does the 17-4 SS powder. Even though there seemed to be some CoCr particles smaller than 7 μm, using the Mie vs. the Fraunhofer theory did not seem to make any difference, probably since there were so few CoCr particles smaller than 7 μm (see Fig. 2). The uncertainty in the CoCr powder results, when considering the D values, is larger than it was for the 17-4 SS powder. From these results, one would conclude that the containers/samples for each material type were identical as far as size distribution goes. Figure 3 presents the D(0.1), D(0.5), and D(0.9) results for each of the 15 CoCr containers examined.

#### 3.3.2 X-Ray Diffraction

**Fig. 4:**
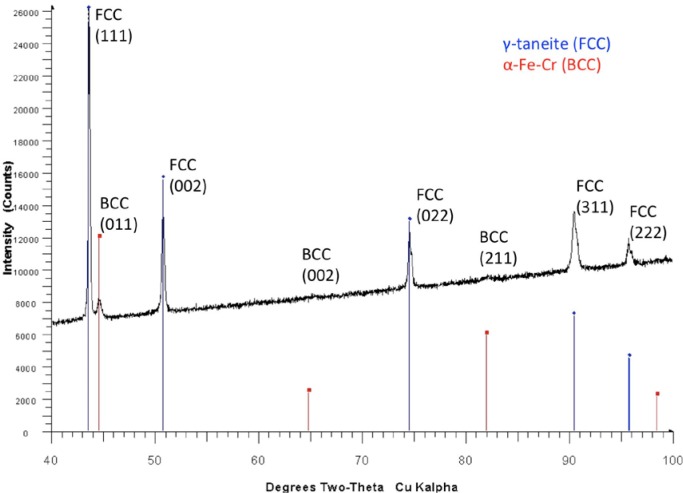
Qualitative XRD scan and phase identification for 17-4SS virgin powder. The scan reveals austenite (δ-FCC, ICDD 33–397) and α Fe-Cr (BCC, ICDD 34-396).

For the 17-4 SS powder, the experimental X-Ray diffraction data shows the material was predominantly composed of austenite, a face centered cubic (FCC) Fe-Ni alloy, and a body centered cubic (BCC) Fe-Cr alloy, with the austenite having the higher volume fraction. Note that this result is not necessarily indicative of the particle interiors. No differences were seen between the four samples of virgin powder, to within experimental uncertainty. Figure 4 shows an example qualitative XRD scan and phase identification for 17-4SS virgin powder. The scan reveals austenite ((δ-FCC, International Centre for Diffraction Data (ICDD) database entry 33–397) and α Fe-Cr (BCC, ICDD database entry 34–396). These results agree favorably with those presented elsewhere [27] for similar metal powders. The height and width of the peaks do not correspond to relative volume fractions – a Rietveld analysis is necessary to obtain this information even semi-quantitatively.

**Fig. 5:**
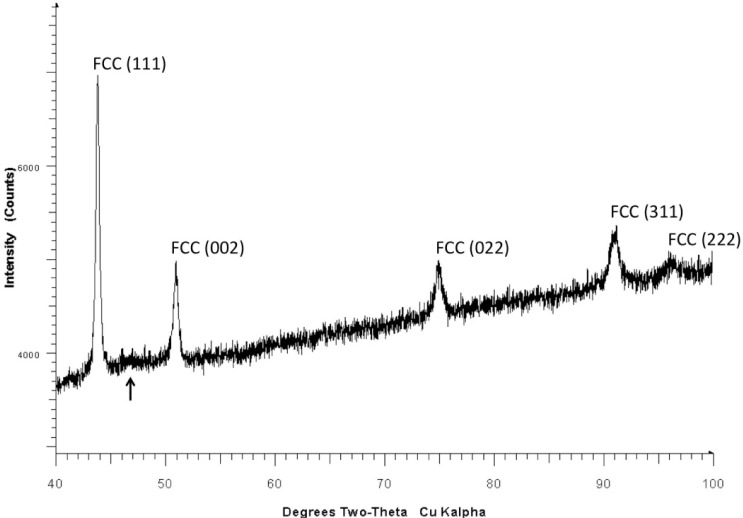
CoCr sample 1 XRD with FCC peak identification and an arrow marking the likely presence of a second phase (denoted as the HCP phase).

For the CoCr powder, the diffraction patterns were consistent with a FCC chromium cobalt nickel molybdenum alloy (ICDD database entry 35–1489). Some very weak, broad diffraction peaks suggest a small secondary phase, denoted HCP (hexagonal close packed). No differences were seen between the 15 samples of virgin powder, to within experimental uncertainty. Figure 5 shows a representative result.

#### 3.3.3 X-ray Photoelectron Spectroscopy

Only one sample of the 17-4 SS powder was examined via XPS – because the XPS measurement takes significant time to perform – so no conclusion could be reached about the similarity of the four vials. This data point, however, is used as a baseline later in the paper to look at recycled powder characteristics via XPS. The cobalt chromium powder is examined next. Four samples randomly selected from the 15 available were chosen to be studied.

An XPS survey scan was first used to determine the elemental concentrations of species present in the cobalt chromium metal specimens (Table 5). The results show the presence of cobalt, chromium, manganese, molybdenum, oxygen, carbon, and silicon on all specimens. The atomic percentages have an uncertainty of about 5 % of the number listed [22]. The XPS spot size for survey mode is about 1 cm^2^. Therefore, the four powders were not identical but were close in composition, at least on the surfaces, which is what XPS scans.

High resolution XPS spectra were then collected for all elements present on the samples. High resolution XPS spectra allow for the determination of the chemical species present in the specimens. The XPS spectra are then curve-fit using literature values for the binding energy (BE) and the full width at half maximum (FWHM) for the various chemical species, in this case pure metals and possible metal oxides. Chemical speciation of the metal powder materials revealed the general trend of high concentrations of metal oxides in addition to small quantities of metal species.

**Fig. 6:**
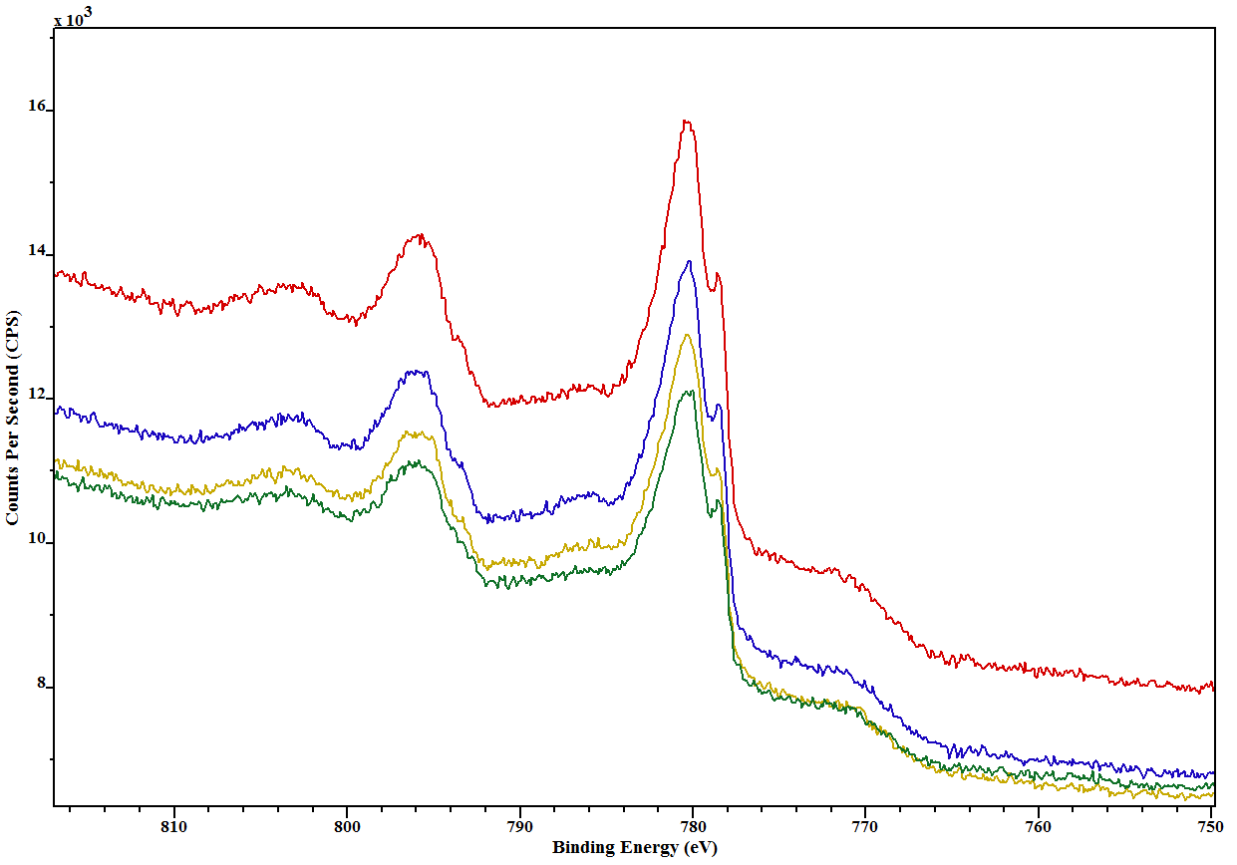
Co 2p XPS spectra for cobalt chromium metal particles CoCr #4 (red), CoCr #6 (green), CoCr #8 (blue), and CoCr #10 (yellow).

Cobalt was present on all samples and the Co 2p XPS spectra shown in Fig. 6 reveal that all samples had similar cobalt species as indicated by the similar shape and intensity of all peaks. Note that the ionization for the 2p shell of cobalt results in an XPS spectrum with two peaks or a doublet corresponding to its electron emission. The peak at the lower binding energy (rightmost) is defined as 2p_3/2_ and the peak at higher binding energy (leftmost) is defined as 2p_1/2_. All powder specimens appear to have combinations of cobalt metal and cobalt oxides.

The Co _2p3/2_ peak was used to determine the concentrations of the chemical species present on the metal powder surfaces. Values for the metal and various metal oxide BE and FWHM were obtained from a standard reference [28].

Three cobalt species, which consisted of cobalt metal (778 eV) and cobalt oxide species (780 eV and 785 eV), were used in the curve-fit process. Typical cobalt oxide species could be CoO (780.4 eV), CoOOH (780.3 eV), Co (OH)2 (781.3 eV), or CoSO4 (784 eV). The results are shown in Table 6. The uncertainties in these data are from the inherent instrument uncertainty as well as uncertainties in curve fitting, for a total uncertainty of about 5 % of the values shown.

**Fig. 7:**
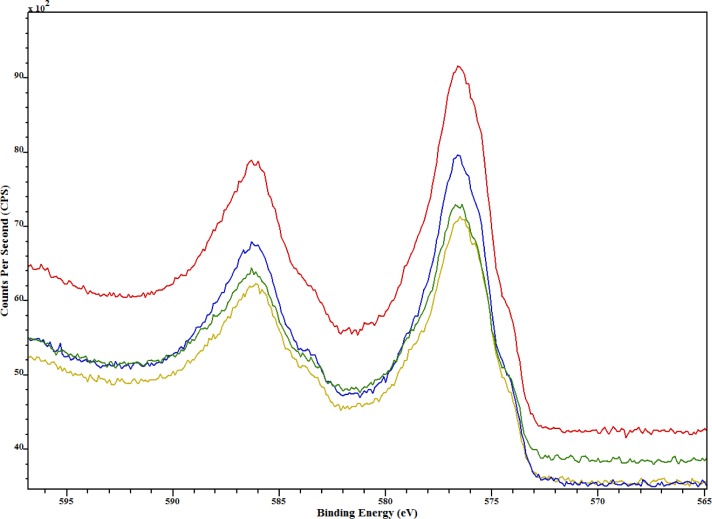
Cr 2p XPS spectra for cobalt chromium metal particles: CoCr #4 (red), CoCr #6 (green), CoCr #8 (blue), and CoCr #10 (yellow).

Chromium was present on all samples and the Cr 2p XPS spectra shown in Fig. 7 reveal that all samples have similar chromium species by the comparable shape and intensity of all peaks. The Cr 2p_3/2_ peak was used to determine the concentrations of the chemical species present on the metal powders. Values for the metal and various metal oxide BE and FWHM were obtained from [28]. Three chromium species, which were chromium metal (575 eV) and chromium oxide species (576.5 eV and 579 eV), were used in the curve-fit process. Two typical chromium oxide species are Cr_2_O_3_ (576.6 eV) and CrO_3_ (580.1 eV).

#### 3.3.4 X-Ray Computed Tomography

X-ray computed tomography was used to examine the size and morphology of the 17-4SS and CoCr powders. For the 17-4 SS powder, a total of 127 000 particles were analyzed from the four samples. The computed particle size distributions agree generally with the laser diffraction results and so will not be reported in this paper. A simple way to analyze particle shape is to define the mutually orthogonal length, L, width, W, and thickness, T, for each particle. These are the dimensions of a rectangular box that just encompasses the particle, with L > W > T for each particle. Then the independent ratios L/W and W/T are effective aspect ratios for the particles. Of course, for a sphere or a cube, these aspect ratios would be unity. The software defines these quantities for each particle and then distributions can be made for each sample.

**Fig. 8:**
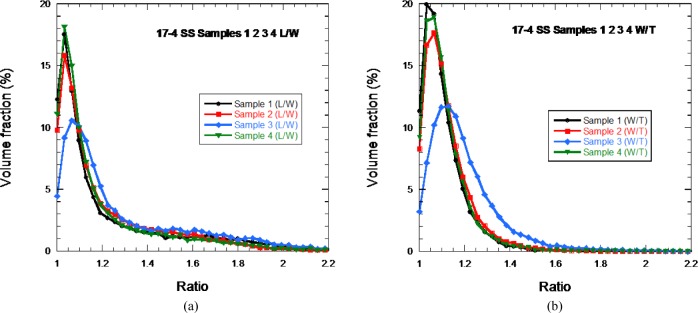
The distribution of (a) L/W and (b) W/T for the four 17-4 SS samples. The solid lines are only guides to the eye.

Figure 8 shows the distributions for L/W and W/T for each of the four 17-4 SS samples. With perhaps the exception of sample 3, the shape distributions are identical within experimental uncertainty, which is estimated to be about 5 % for each ratio for each particle. The distributions show that the particles were definitely not uniform spheres, though the peaks in the distributions were near unity. As can be seen in the SEM images in Fig. 10, there are very non-spherical particles, while others, which have a small particle attached to a larger one, would also have an L/W ratio greater than unity.

**Fig. 9:**
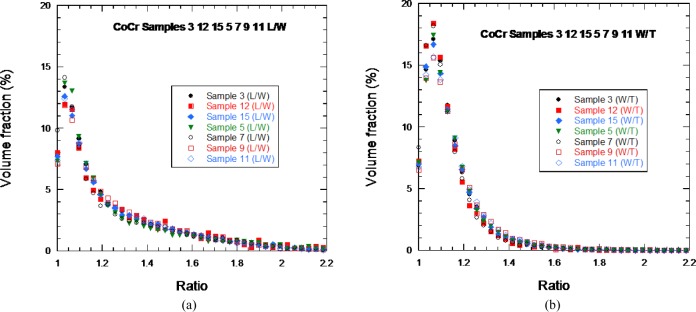
The distribution of (left) L/W and (right) W/T for seven of the 15 CoCR powder samples.

Seven CoCr samples were analyzed, with a total of 197 000 particles studied. Figure 9 shows the shape distributions for these particles, similar as in Fig. 8, for the 17-4 SS particles. Again, these particles were not simple spheres.

#### 3.3.5 Scanning Electron Microscopy

**Fig. 10:**
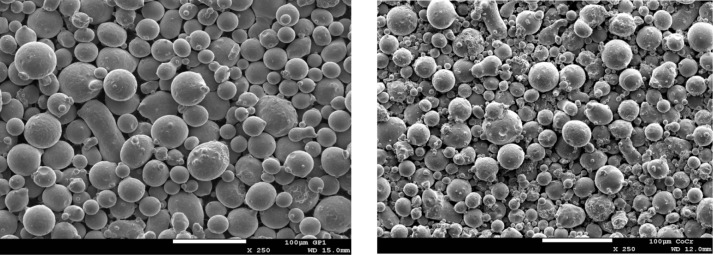
SEM micrographs of (left) 17-4 stainless steel and (right) CoCr powder. The white scale bars represent a length of 100 μm.

Samples from the raw, sieved and unsieved builds, and sieve residue powders were imaged by scanning electron microcopy using a consistent imaging scheme to facilitate comparison. Figure 10 shows representative SEM micrographs for the 17-4 stainless steel and CoCr powders. The white scale bar represents 100 μm. Although many particles seem quite spherical, one can visually identify non-spherical particles, mainly caused by smaller spherical particles being attached to larger spherical particles. This was indicated in Figs. 8 and 9, with W/T ratios equal to or larger than unity. However, in Fig. 10 there are also single particles that are non-spherical without any other attached particles. Also, the images show that the CoCr powder has many more small particles, in accord with the laser PSD results. The appendix includes many more SEM images of the two powder types.

### 3.4 Recycling

In what ways, if any, does virgin powder change after being exposed to and recycled from one or more additive manufacturing build cycles? There are varying industry practices for how many times a powder can be reused, but these have little or no scientific rigor to support them. An experiment was designed to see what physical and chemical changes occur to 17-4SS powder that has been exposed to and recycled from one or more additive manufacturing build cycles. In a DMLS build, a powder is poured into the hopper, and then repeatedly deposited onto the build bed and selectively melted to make the part. When the build is completed, the unused powder is recycled by being collected and brushed from the build chamber using a soft-bristled brush and then passed through an 80 μm opening sieve. The powder passing through the sieve is put back in the hopper, and any +80 μm sieve residue is discarded.

A set of eight builds was performed. Starting with virgin powder, after each build was completed a powder sample was taken from around the completed part before collecting the powder. Another sample was the taken after collection and sieving. In addition, the sieve residue after the first build was retained for further study. These 17 samples were systematically characterized using the various techniques described in Sec. 2.

#### 3.4.1 Laser PSD

**Fig. 11:**
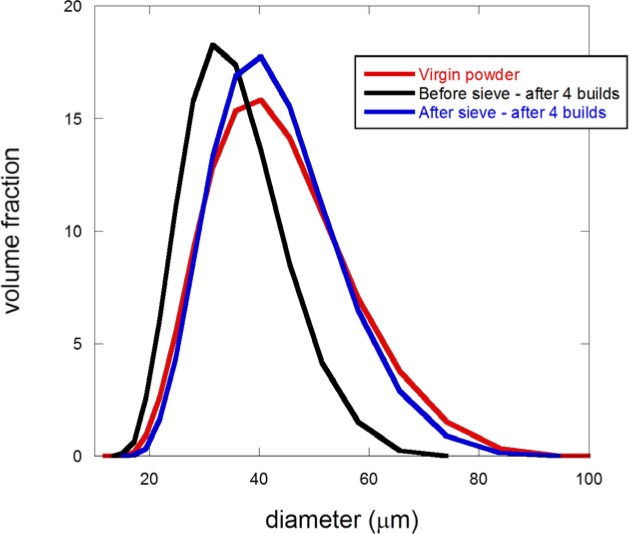
Particle size distributions (PSD) of 17-4 stainless steel powder measured by laser diffraction for virgin powder, and recycled powder after 4 builds, both before and after sieving.

Figure 11 shows the particle size distribution (PSD), as measured by laser diffraction, for virgin powder, and powder reclaimed after four builds. The PSD for the reclaimed powder was measured for the powder collected both before and after sieving (80 μm sieve opening). Comparing the three curves in Fig. 11, it is first interesting to see that the PSD curves for the virgin powder and the powder after sieving are almost identical. However, the powder sampled after 4 builds but before sieving is shifted to the left, absent of particles that have measured diameters greater than about 65 μm. The first result is reassuring – the powder that has been reclaimed and processed via sieving, ready to be used in a new build, has essentially the same PSD as the virgin powder. However, the second result is quite puzzling – how can sieving – a process for removing larger particles – apparently restore missing larger particles?

The answer to this problem comes when one considers the manufacturing process and the powder characteristics. The powder PSD extends roughly between 10 μm and 80 μm. The vertical distance between sweeps of the recoating arm is about 20 μm. While some of the larger particles will settle into the powder bed, most of the larger particles will be carried away by the recoating arm and pushed towards the side. Therefore, the used powder bin would be enriched with the larger particles, and the powder around the part, from where pre-sieving samples were exclusively taken, will be depleted with respect to the larger particles. Upon examining the sampling data, it was found that all samples of reclaimed powder that were taken after a build but before sieving were taken from around the part, the powder region that would be expected to be depleted in larger particles. This is the reason for the appearance of this curve in Fig. 11. However, after a build was completed, all the remaining powder was reclaimed and passed through an 80 μm sieve, thereby restoring the larger particles, which then again make their appearance in Fig. 11. A similar result was found for every reuse cycle investigated, and the findings from the X-ray CT results also confirmed these results and their interpretation. These laser diffraction size analyses and sampling results give insight into the details of the manufacturing process – the larger particles were essentially not incorporated into the process.

**Fig. 12:**
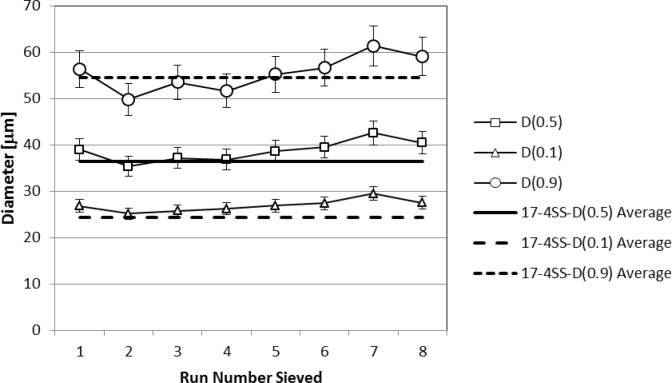
Evolution of the PSD characteristics of the samples for each run after sieving.

Figure 12 shows the values of D(0.1), D(0.5), and D(0.9) for the eight reclaimed powders after sieving. Recall that D(α) is defined such that D is the diameter, as measured by LD, for which α is the mass or volume percent (or fraction) of particles with measured diameter less than D. In Fig. 12, these values are increasing, especially for builds 5 through 8. Why might the reused powder gradually increase in size as the number of reuse cycles increases? The 80 μm sieve puts a limit on the maximum size particle that could be reused, so any increase in size has to come from the smaller particles. If, for example, two 30 μm particles became partially sintered, they would still pass through the sieve but LD would measure them as a larger particle. The probability of these kinds of events happening would be the same for each build, since it could only occur at the part’s interface with the unsintered powder, where a high enough temperature for sintering (typically 2/3 the melting temperature) could be present. However, the number of these kinds of multi-particles would then increase with build number, precisely as is seen in Fig. 12.

#### 3.4.2 Physical and Chemical Changes During 17-4 Eight Build Experiment

**Fig. 13:**
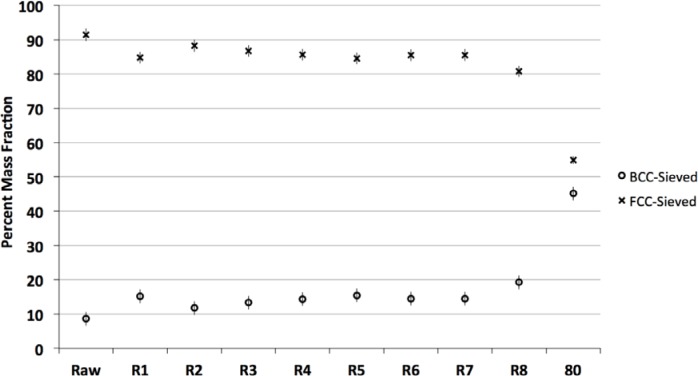
Quantitative XRD results for relative phase proportions of FCC and BCC phases for the 17-4 SS sieved powders after each of eight builds compared to the raw (far left) and +80 sieve residue (far right) indicates an increase in the BCC phase with build. Uncertainty bars are from three replicates. Raw = virgin powder and R# is the number of the build.

Quantitative X-ray diffraction was performed on the powder from all eight builds, always after sieving. Results demonstrated that the 17-4 SS powder was composed of a face-centered-cubic (FCC) Fe-Ni alloy (generic name of austenite), and a body-centered-cubic (BCC) α Fe-Cr alloy. Since the XRD results are sensitive to mainly the first few micrometers of these large (compared to usual XRD powders) particles, these results could be surface-sensitive. Figure 13 shows the progression of the volume fraction of these two phases. There is a small increase in the volume fraction of the BCC phase along with a corresponding decrease in the FCC phase. This is possibly due to the extra heat that some of the reused powder sees near the part edges. The point marked “80” is from the SS powder that was retained on the sieve and will be discussed later. The uncertainty bars in Fig. 13 represent the standard deviation from three replicates.

#### 3.4.3 X-Ray CT Shape Results During 17-4 Eight Build Experiment

Samples consisting of 17-4 SS powders dispersed in epoxy were made with powder reclaimed after 1, 4, and 8 builds, both sieved and unsieved. Similar to Sec. 3.3.4, once the particles were characterized in the X-ray CT, the length (L), width (W), and thickness (T) were calculated for each kind of sample and a distribution constructed in terms of volume fraction of the total powder sample considered.

**Fig. 14:**
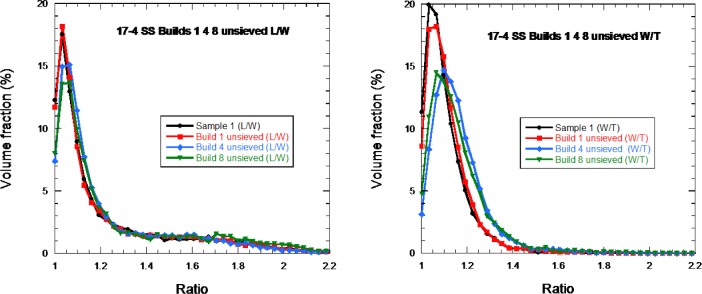
Particle shape distribution for virgin, 1-build, 4-build, and 8-build 17-4 SS unsieved powders: (left) L/W distribution, (right) W/T distribution in terms of percent of total powder volume fraction.

The results of Fig. 14 show that there is no significant change in the L/W ratio distribution from 1 build to 8 builds, but there is some change in the W/T ratio going to 4 builds and 8 builds. The reason for this will be studied further but is unknown at present.

#### 3.4.4 XPS Results During 17-4 SS Eight Build Experiment

XPS was used to determine the elemental concentrations of species present in one of the virgin stainless steel specimens (Table 8). The results show the presence of copper, iron, chromium, nickel, oxygen, carbon, and silicon on all specimens. This procedure was also carried out for the recycled material, after sieving and taken after 2, 5, and 8 build cycles. Table 8 shows that there were no significant differences in elemental concentrations between the virgin and recycled powder materials.

High resolution XPS spectra were then collected for all elements present on the samples, but only the majority element, iron, is presented below. High resolution XPS spectra allow for the determination of the actual chemical species present in the specimens. The XPS spectra are then curve-fit using literature values for BE and FWHM for the various chemical species, in this case pure metals and possible metal oxides [28]. Chemical speciation of the metal powder materials revealed the general trend of high concentrations of metal oxides in addition to small quantities of metal species.

**Fig. 15:**
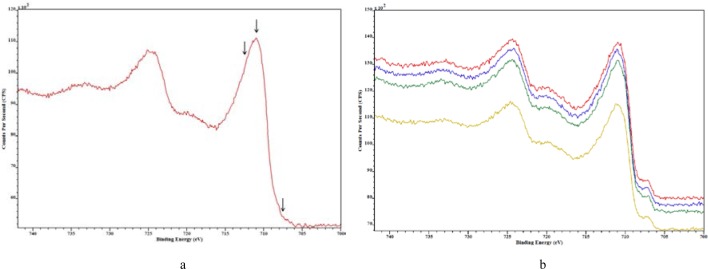
Fe 2p XPS spectra for stainless steel particles: a) residual sample after sieving, b) 17-4 SS sample #2 (red), Recycle-2 builds (blue), Recycle-5 builds (yellow) and Recycle-8 builds (green). Arrows in a) indicate the position of the various iron oxide species used in curve-fitting the spectrum.

Iron was present on all samples and the Fe 2p XPS spectra shown in Fig. 15 reveal that all samples have similar iron oxide species regardless of recycling the powder material as indicated by the similar shape and intensity of all peaks. The Fe 2p_3/2_ peak was used to determine the concentrations of the chemical species present on the metal powders. Values for the metal and various metal oxide BE and FWHM were obtained from Ref. [28]. Three iron species, which consisted of pure metal (707 eV) and metal oxide species (710 eV and 712 eV), were used in the curve-fit process. Several possible metal oxide species are: FeO (709.6 eV), Fe2O3 (710.9 eV), and FeS (711.9 eV).

Table 9 shows that the concentration of species was not significantly different between the virgin and recycled powder materials. The residual sample showed no presence of iron metal. The oxide species are separated into two peaks at 712 eV and 710 eV for simplicity.

### 3.5 Analysis of Sieve Residue

After each build, the spread-but-unused powder is collected and passed through an 80 μm sieve. In these experiments, after the first build, the powder that did not pass through the sieve (sieve residue) was collected and analyzed. This powder, even though it is not going to be included in the actual build, is of interest since the only way that particles can become large enough to not go through the sieve is to become multi-particles. That means these excluded particles must come from near the built part boundaries, where stray energy from the laser can produce enough heat to partially sinter or melt particles together. Analyzing the sieve residue then gives information about how particles are affected near the boundaries, which might go through the sieve and be re-used.

Three kinds of particles could be in this residue. The first are particles larger than 80 μm that were present in the original virgin powder – some particles of this size were seen in the laser diffraction results. Note that the original virgin 17-4 SS powder was not sieved before the first build was carried out. Secondly, the unused powder is swept into the sieve using a soft bristle brush, so some of these bristles could separate from the brush and end up in the sieve – this was seen. Finally, two or more particles that separately were smaller than 80 μm could become lightly sintered if they experienced some of the laser’s heat and so might not pass through the sieve. Because at least some of the particles could experience high heat, the possibility exists of phase changes, at least at the particle surfaces, to which the XRD technique is sensitive. In this section, results from SEM, XRD, and X-ray CT are presented. Note that laser diffraction was not performed – there was not enough sieve residue powder recovered to allow this technique, along with all the others, to be performed. More such powder is planned to be saved in future experiments. Since the laser diffraction particle size measurement assumes spherical particles, and the sieve residue are almost all aggregates of near-spherical particles, the “size” that will be measured will only be an equivalent sphere diameter.

**Fig. 16:**
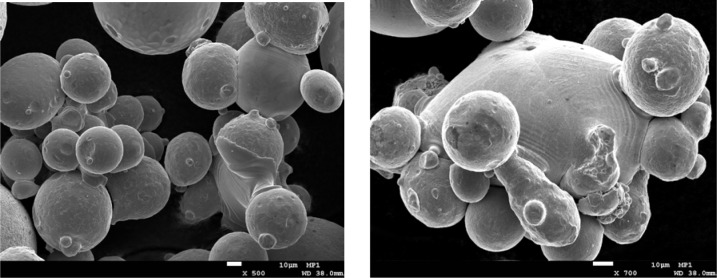
SEM images of 17-4 SS sieve residue powder where multi-particles are seen.

Figure 16 shows two SEM images of some of the 17-4 SS sieve residue powder. Notice the many multi-particles in the left-hand image, and the multi-particle particle in the right-hand image, which shows signs of a melt phase being formed. Other multi-particles are partially sintered.

**Fig. 17:**
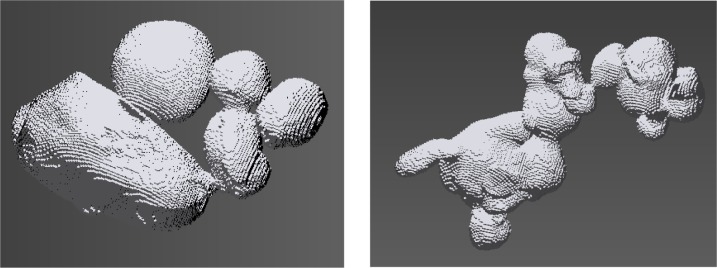
Two 3-D images of multi-particles captured by X-ray CT analysis. The voxel structure of the original images can be seen.

Figure 17 shows two 3-D images from multi-particles that were captured by the X-ray CT. No shape analysis was possible for these particles, since the spherical harmonic analysis process requires the particles to be star-shaped [16]. Very large particles are possible, since many single particles can be partially sintered into a larger one.

X-ray diffraction was used to examine the composition of the sieve residue powder. Figure 13 showed the result in the point marked “80”. The FCC (austenite) component of the powder decreased greatly in volume fraction, down to about 55 %, while the BCC component increased in volume fraction, up to about 45 %. Again, these volume fractions are semi-quantitative and mainly reflect the particle surfaces. But it is clear that the composition of the sieve residue particles, due to their exposure to the high heat of the laser, has changed significantly.

XPS spectra were used to analyze the differences between the virgin 17-4 SS powder and the sieve residue 17-4 SS powder. A survey scan, with results shown in the first two rows of Table 10, showed that the atomic percentages differed widely between the two powders. The sieve residue powder showed lower copper, chromium, and carbon amounts and greater iron and silicon concentrations than the virgin powder.

The second set of data in Table 10, for iron, shows clearly that there was no metallic iron on the surface of the sieve residue powder compared to the virgin powder. This correlates well with the XRD finding of the phase changes on the sieve residue powder surface and is presumably due to the high temperatures that this material has seen – the welding into multi-particles is a clear indication that this happened. The O 1s XPS spectrum for the sieve residue sample showed a very different oxygen spectrum from the virgin material with different peaks and intensities, resulting in different amounts of oxide compounds on the powder surfaces compared to the virgin powder (third set of data in Table 10).

## 4. Conclusions

Several conclusions can be drawn from these analyses:

- Container-to-container consistency. The powder is consistent across the production lot (e.g., the samples from distinct nominally identical containers for both CoCr and 17-4 SS.) The characteristics considered were

1. ○ Size distribution
2. ○ Particle shape
3. ○ Crystallographic form and chemistry
4. ○ Density

- In all the imaging techniques described in this paper, no internal particle porosity was seen, although individual grain boundaries were visible.
- The virgin CoCr powder included more small particles than did the virgin 17-4SS powder. This is shown in the SEM and the laser diffraction PSD. The D(0.5) value for the CoCr powder was D(0.5) = 23 μm, while that for the 17-4SS powder was D(0.5) = 36 μm. The D(0.1) values were: 8.9 μm (CoCr) and 24.4 μm (17-4SS), which quantitatively illustrates the many more small particles for the CoCr powder vs. the 17-4SS powder.
- The predominant 17-4SS chemical phase was FCC austenite, with a minor BCC component. The main CoCr component was FCC, with a potential trace of a HCP crystal structure.
- Particles are roughly spherical, but with important non-sphericities due to fracture, satellites, joined particles, and different intrinsic morphology (e.g., “tear-drop” shape). This was quantitatively illustrated vs. distribution functions for the L/W and W/T aspect ratios.
- Recycling increases: powder size distribution with progressive builds.
- The stainless steel powder in the sieve residue show characteristics of melting and particle joining. The sieve residue materials have a distinctly different proportion of FCC to BCC phases, where the FCC decreases and BCC increases compared to the virgin pwder.
- XPS indicated significant differences in the surface chemistry of the SS sieve residue particles vs. the virgin 17-4 SS particles.
- Differences in particle size distribution, as measured both by laser diffraction and by X-ray CT, from samples taken before and after sieving, showed that the spreader arm was definitely preferentially transporting larger particles (> 60 μm) past the build plate. These particles were then selectively not incorporated into the build. Hence, careful particle size measurements gave insight into the manufacturing process used.

## 5. Future Work Needed

In future work, the powder recycling experiment will be repeated and improved by taking samples at several points around the building part, including where larger particles are thought to be deposited and the sieve residue. The virgin powder particle size distribution will be systematically varied, and the interaction of the PSD with the process and the tensile strength of the built part (tensile dogbone specimens) will be determined. Complete powder characterization will again be carried out with the sophisticated techniques demonstrated in this paper, but will also be compared with industrial standard techniques to see how these can/should be improved so as to capture essential powder parameters.

## Figures and Tables

**Table 1:** Metrology methods used for powder characterization in this paper and their associated measurands

Measurand	Metrology Method
Density of particles	Helium Pycnometry
Particle Size Distribution	Laser Diffraction
Particle Size and Morphology	X-Ray Computed Tomography
Particle Crystalline Phases	X-Ray Diffraction
Particle Morphology	Scanning Electron Microscopy
Particle Elemental Composition	Energy Dispersive Elemental Analysis
Particle Surface Molecular/Chemical Composition	X-Ray Photospectroscopy

**Table 2:** Helium pycnometry results for the density of virgin powders

Powder type	Powder density (g/cm^3^)
Sample 1 – 17-4 SS	7.878 ± 0.005
Sample 3 – 17-4 SS	7.875 ± 0.005
Sample 4 – CoCr	8.305 ± 0.005
Sample 9 – CoCr	8.301 ± 0.005
Sample 14 – CoCr	8.304 ± 0.005

**Table 3:** Numerical PSD results for 17-4SS containers

Sample name			
	
	D(0.1)	D(0.5)	D(0.9)
	
17-4SS #1	24.7	36.7	54.6
17-4SS #2	25.0	36.6	53.4
17-4SS #3	24.0	36.3	54.9
17-4SS #4	23.8	36.3	55.2

*average*	*24.4*	*36.5*	*54.5*
*STD*	*0.6*	*0.2*	*0.8*

**Table 4:** Distribution characteristics of cobalt chrome samples from containers #1–15, all taken from the same production heat lot

Sample	D(0.1)	D(0.5)	D(0.9)
1	9.0	22.1	42.8
2	9.3	24.3	46.5
3	8.8	23.0	44.7
4	8.9	23.4	45.8
5	9.0	23.3	45.2
6	9.3	24.3	47.0
7	8.4	21.6	43.3
8	9.6	24.3	46.1
9	8.9	23.2	45.4
10	8.7	21.9	42.5
11	8.8	22.9	44.6
12	8.9	23.6	45.5
13	9.6	24.0	45.5
14	8.8	22.3	43.5
15	8.1	21.6	42.0

***average***	8.9	23.0	44.7
***STD***	0.40	0.96	1.5

**Table 5:** Atomic percentage concentrations observed in XPS survey scan for cobalt chromium particles

Sample	Co 2p	Cr 2p	Mn 2p	Mo 3d	O 1s	C 1s	Si 2p
***Sample 4 – CoCr***	7.4	6.8	2.3	1.4	34.5	37.1	10.6
***Sample 6 – CoCr***	5.2	5.0	1.2	0.9	28.3	50.9	8.5
***Sample 8 – CoCr***	8.1	7.3	2.7	1.3	35.5	34.8	10.3
***Sample 10 – CoCr***	7.5	5.4	2.4	1.0	30.3	44.2	9.9

**Table 6:** Cobalt species concentration obtained from curve-fitting Co 2p_3/2_ XPS spectra. The units are arbitrary, and the values should only be used to compare with each other.

	Cobalt species concentration		
	785 eV	780 eV	778 eV
***Sample 4 – CoCr***	57.7	978	77.7
***Sample 6 – CoCr***	50.3	595.6	92.6
***Sample 8 – CoCr***	91.6	791.3	147.3
***Sample 10 – CoCr***	69.2	700	122.7

**Table 7:** Chromium species concentration obtained from curve-fitting Cr 2p_3/2_ XPS Spectra. The units are arbitrary, and the values should only be used to compare with each other.

	Chromium species concentration		
	*579 eV*	*576.5 eV*	*575 eV*
***Sample 4 – CoCr***	111.1	1084.9	39.2
***Sample 6 – CoCr***	111.1	747.8	27.9
***Sample 8 -CoCr***	111.1	975.8	33.6
***Sample 10 – CoCr***	111.1	821.8	29.9

**Table 8:** Atomic percentage concentrations observed in XPS survey scans for 17-4 SS powders

	Cu 2p	Fe 2p	Cr 2p	Ni 2p	O 1s	C 1s	Si 2p
***17-4 SS sample #2***	3.5	5.8	4.5	0.2	40.0	40.8	5.2
***Recycle after 2 builds***	3.9	7.3	4.4	0.2	42.1	37.1	5.1
***Recycle after 5 builds***	2.9	4.7	3.4	0.0	38.1	46.5	4.4
***Recycle after 8 builds***	3.3	5.2	4.3	0.0	41.5	40.6	5.1

**Table 9:** Iron species concentration obtained from curve-fitting Fe 2p_3/2_ XPS spectra. The units are arbitrary, and the values should only be used to compare with each other.

	Iron species concentration		
	*712 eV (Fe oxide)*	*710 eV (Fe oxide)*	*707 eV (Fe)*
***17-4 SS sample #2***	609.6	325.6	44.2
***Recycle after 2 builds***	554.5	361.1	45.3
***Recycle after 5 builds***	466.5	273.2	33.6
***Recycle after 8 builds***	523.4	367.8	39.2

**Table 10:** Atomic percentage concentrations observed in XPS survey scans for 17-4 SS powders. Iron and oxygen species concentrations obtained from curve-fitting – the units are arbitrary, and the values should only be used to compare with each other.

	Cu 2p	Fe 2p	Cr 2p	Ni 2p	O 1s	C 1s	Si 2p
***17-4 SS sample #2***	3.5	5.8	4.5	0.2	40.0	40.8	5.2
***Residual***	0.7	8.2	1.9	0.0	44.1	27.8	17.2
***Iron***	***712 eV (Fe oxide)***		***710 eV (Fe oxide)***		***707 eV (Fe)***		
***17-4 SS sample #2***	609.6		325.6		44.2		
***Sieve residue***	814.8		239.7		0		
***Oxygen***	***534 eV***	***533 eV***	***532 eV***	***531 eV***	***530 eV***		
***17-4 SS sample #2***	504	943	3435	2028	4675		
***Sieve residue***	2722	0	3579	2861	2473		

**Fig. A1:**
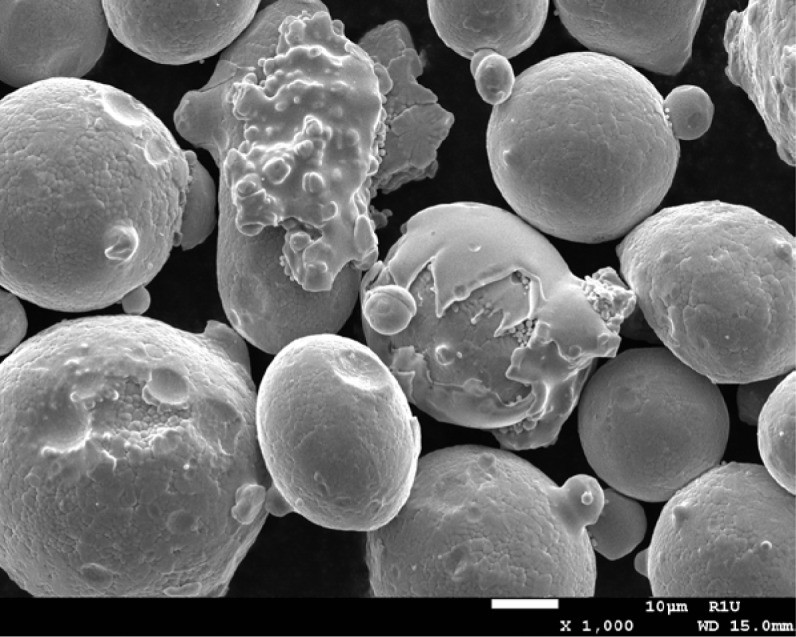
Rounded, spherical powder particles for 17-4SS virgin powder. This image shows accretionary coatings affecting powder roundness, pocks (golfball texture), and nubs. A smooth surface is seen in the centered-accretionary particle, while the individual material phases are seen clearly in the left-most particles as orange-peel like structure. The small white line in the figure is 10 μm long (“10 μm” is printed to the right of the white bar).

**Fig. A2:**
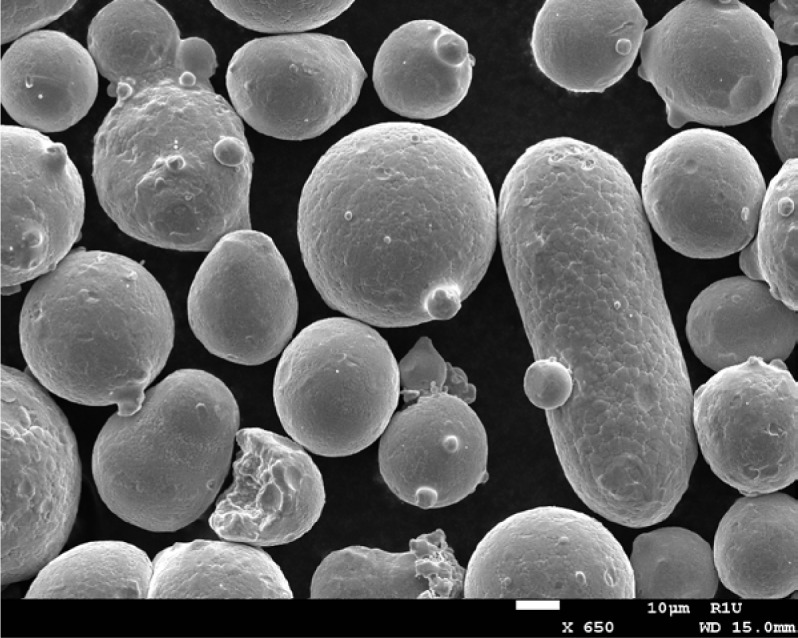
17-4SS virgin powder, showing elongated powder particles, bonded particles, and evidence of fracture.

**Fig. A3:**
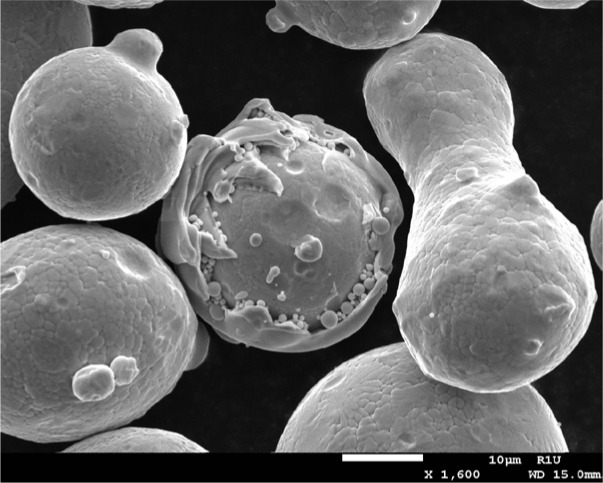
17-4SS virgin powder, showing accretionary particles, bonded/elongated particles, and surface textures of nubs and pocks.

**Fig A4:**
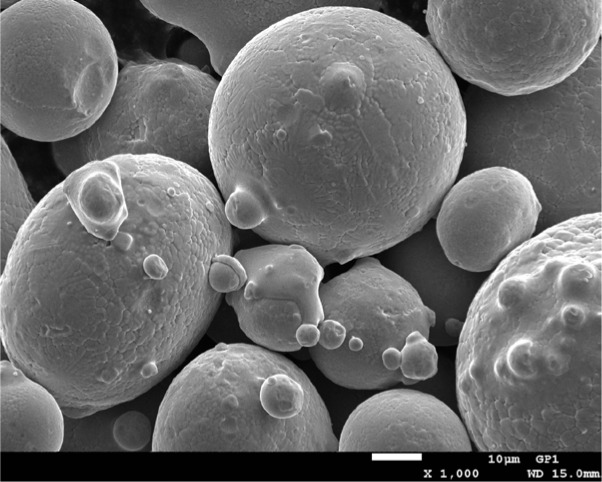
1000x view of virgin 17-4SS powder. Smaller satellites attached to the particles, a common morphology from powders that are produced via gas-atomization, are evident.

**Fig. A5:**
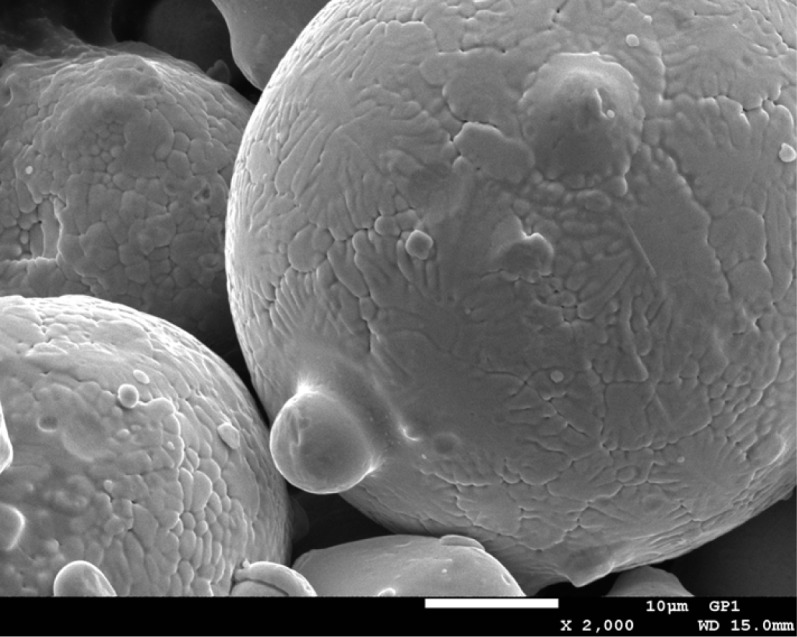
2000x view of part of Fig. A4.

**Fig. A6:**
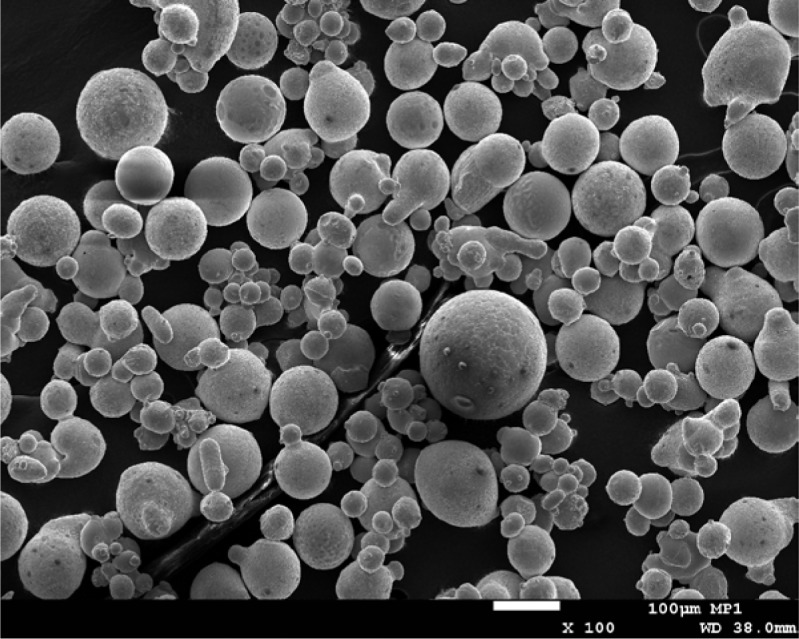
100x magnification of 17-4SS powder mixed with sieve residue.

**Fig. A7:**
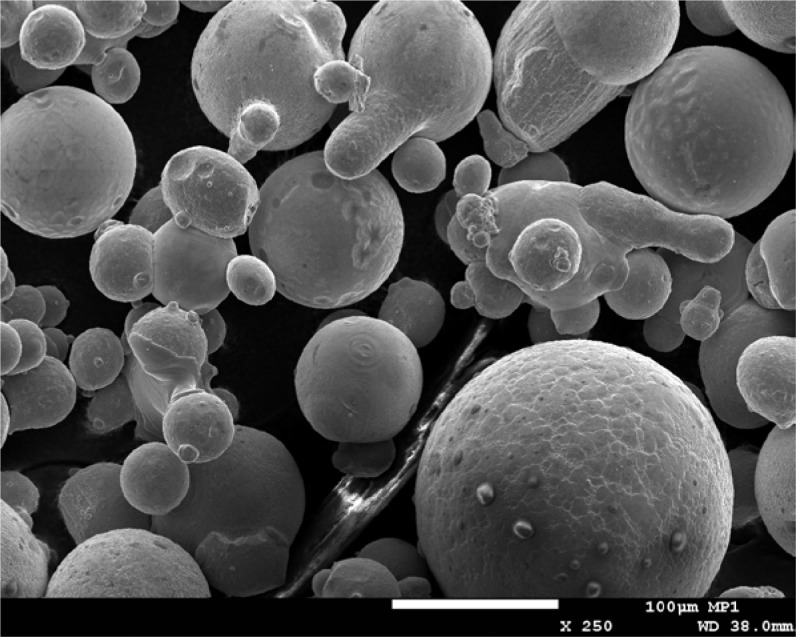
250x view of part of Fig. A6.

**Fig. A8:**
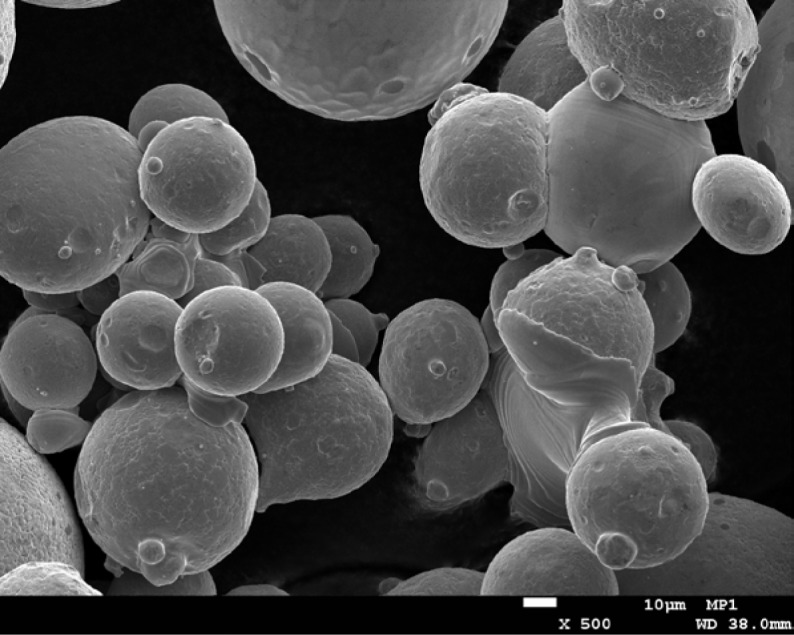
500x view of part of Fig. A7. Note the fused powder particles with some appearing to reflect a melt phase bonding multiple grains. Warped, “etched”, and melted structures are also apparent.

**Fig. A9:**
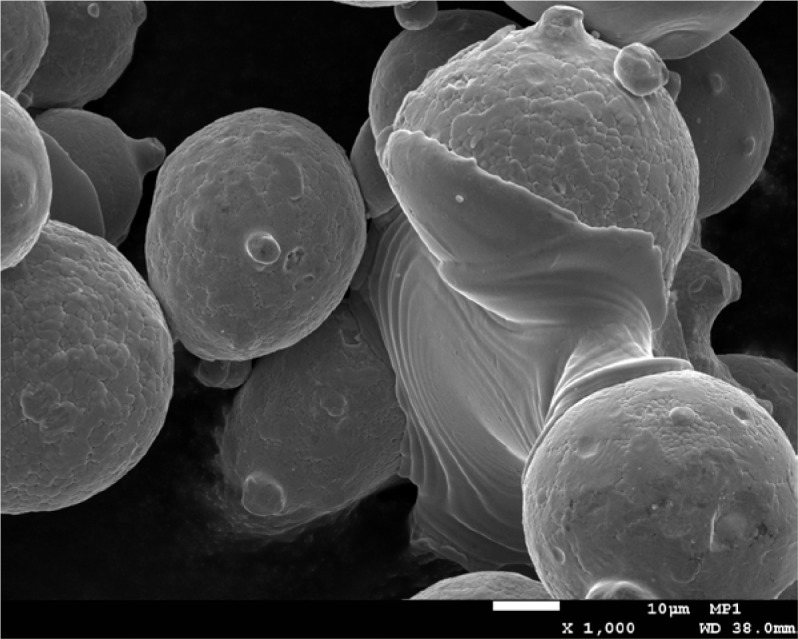
1000x view of part of Fig. A8, showing fused and etched grains.

**Fig. A10:**
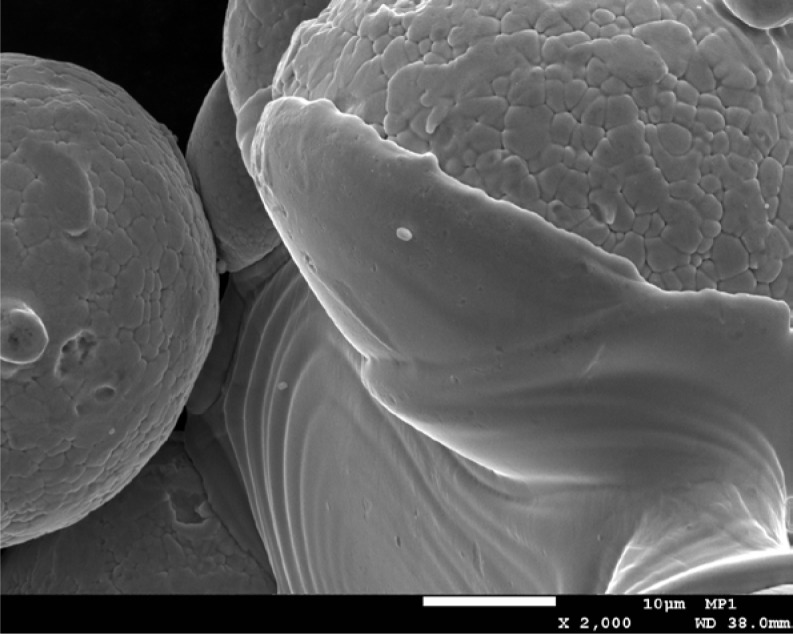
2000x of part of Fig. A9 showing melt bonding of powder particles.

**Fig. A11:**
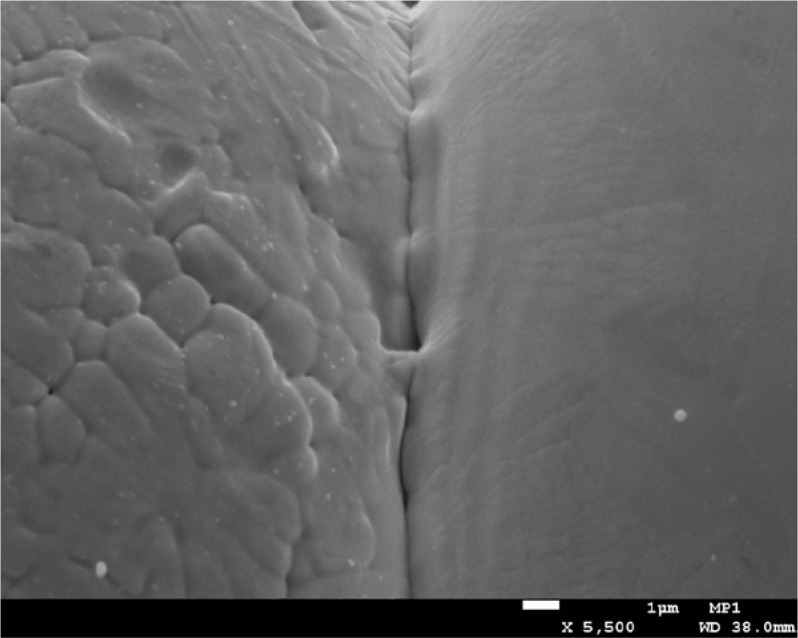
5500x image of contact point between two powder particles taken from Fig. A10. The two grains exhibit two grain textures, boundary-etched (left) and smoothed (right) and display a contact interface between them.

**Fig. A12:**
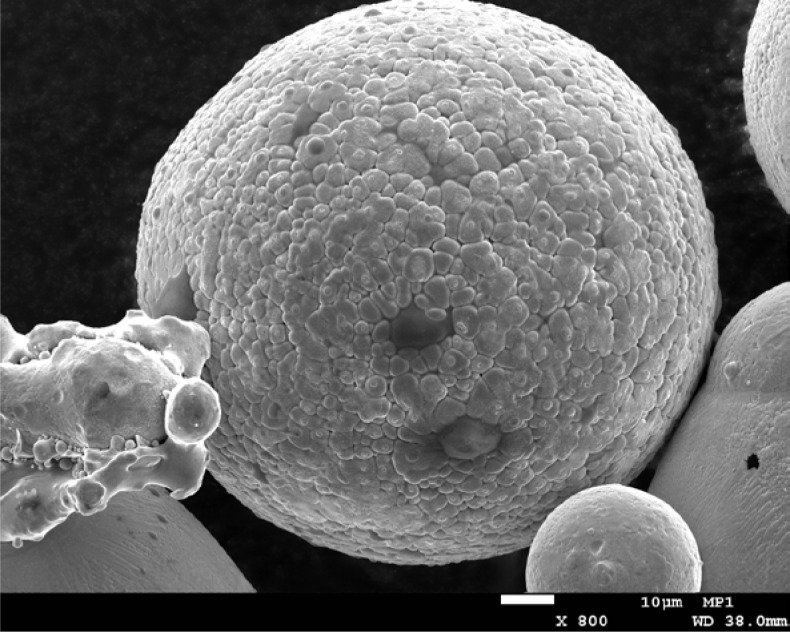
800x view of a sieve residue 17-4SS particle exhibiting a framework grain structure with enhanced grain boundaries.

**Fig. A13:**
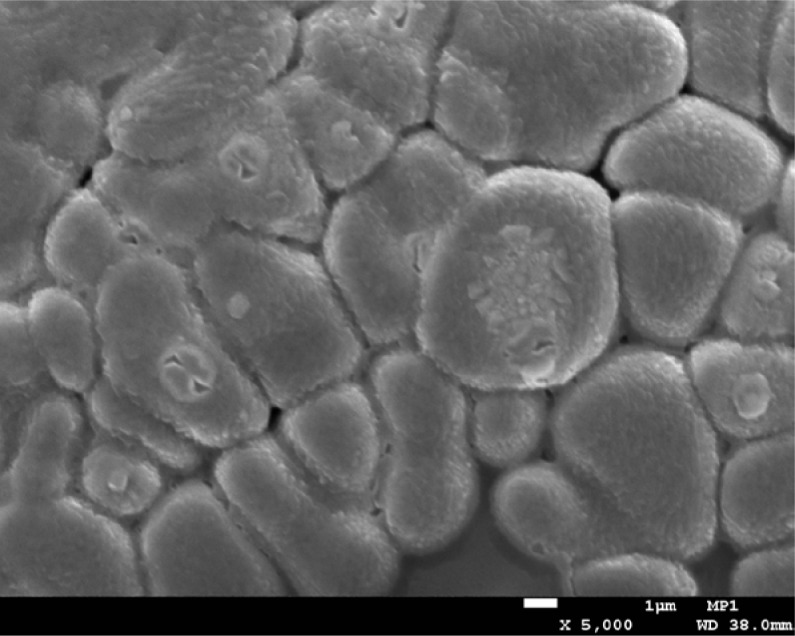
5500x close up of the grain surface texture shown in Fig. A12. Note the enhanced grain boundaries and apparent matrix binding phase.

**Fig. A14:**
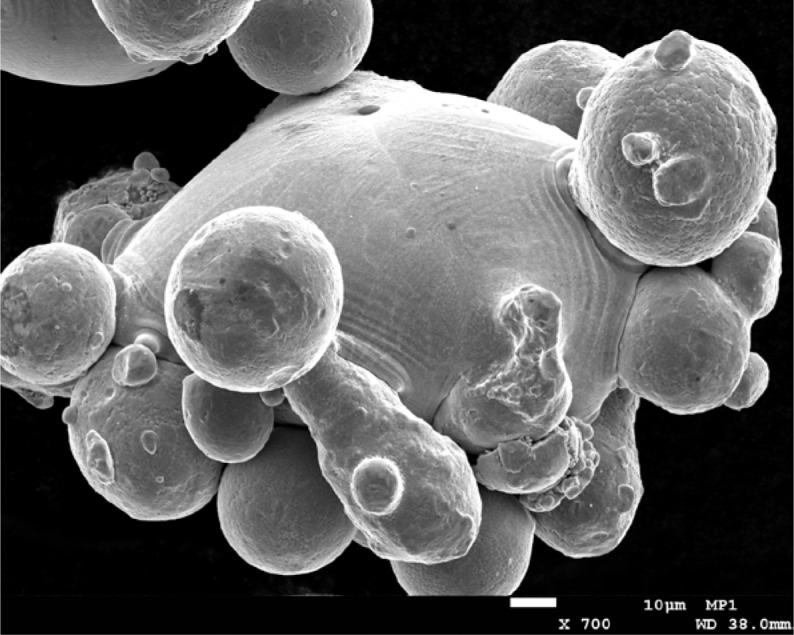
700x view of multiple 17-4SS sieve residue particles that are clearly bonded by a melt phase.

**Fig. A15:**
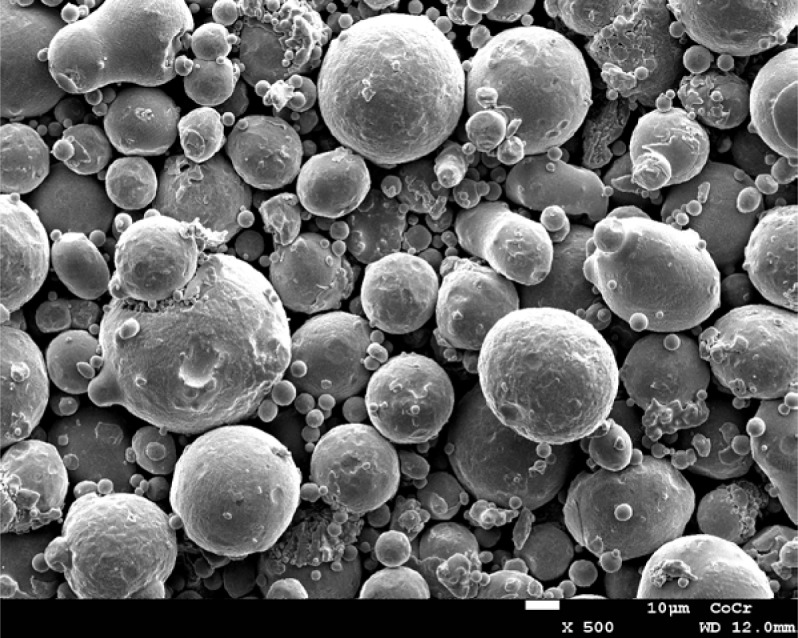
A 500x view of virgin CoCr powder.

**Fig. A16:**
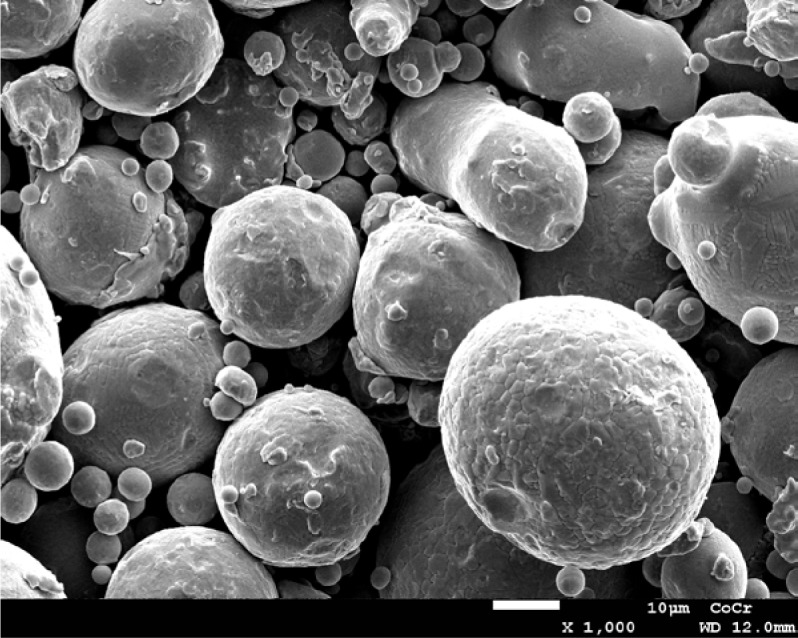
A 1000x view of a portion of the image shown in Fig. A15.

**Fig. A17:**
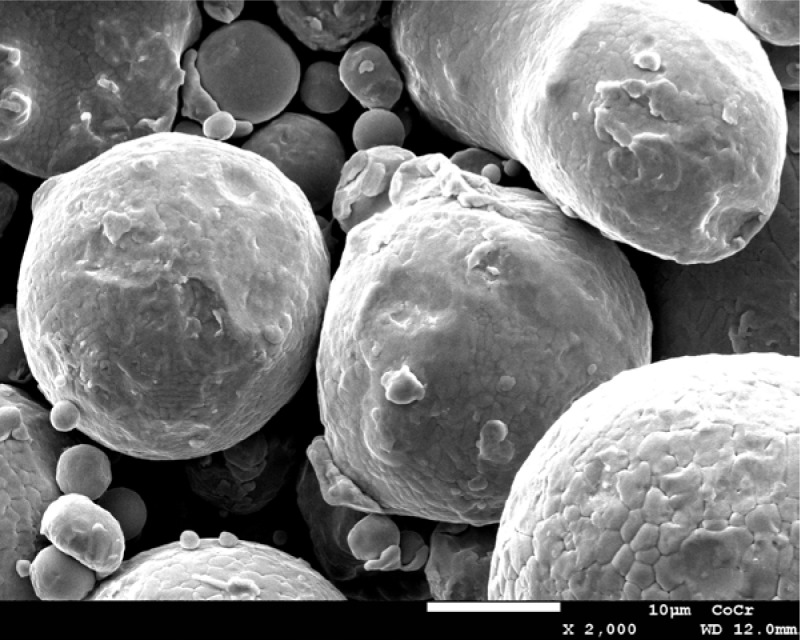
A 2000x view of a portion of the image shown in Fig. A16.

## Footnotes

2 International Centre for Diffraction Data: http://www.icdd.com

3 Strictly speaking, the stainless steel powder should be officially designated as UNS S17400, indicating that this material has this chemical composition. Usually, using 17-4 alone implies precipitation hardening, which is not the case for this powder. In this paper, 17-4 is used as a shorthand notation, in accord with AM customary usage.

## 6. Appendix A: SEM Images of 17-4SS and CoCr Powder Samples

This section compliments Sec. 3.3.5 by showing additional interesting powder morphologies for the 17-4SS and CoCr powders examined in this study.

Figures A1–A5 show views of different virgin 17-4SS particles, where the white bars correspond to a dimension of 10 μm. These images were selected to show several different common shape variations. Figure A6 shows a mix of virgin powder and sieve residue powder – note the many multi-particles, which tend to be made up of the smaller particles. The smaller the particle, the greater the surface curvature and the easier to sinter.

Figures A7–A13 show increasingly magnified views of the particles in Fig. A6. The figure captions point out some of the interesting features.

Figures A15–A17 show increasingly magnified views of some of the CoCr powder particles at a similar magnification to that used in Figures A1–A6. Clearly, the CoCr powders have many more smaller particles at smaller diameters than do the 17-4SS powders. Small particles attached to large particles and particles with other kinds of non-round morphologies are clearly evident.
